# Genetic and environmental influences on structural brain measures in twins with autism spectrum disorder

**DOI:** 10.1038/s41380-018-0330-z

**Published:** 2019-01-18

**Authors:** John P. Hegarty, Luiz F. L. Pegoraro, Laura C. Lazzeroni, Mira M. Raman, Joachim F. Hallmayer, Julio C. Monterrey, Sue C. Cleveland, Olga N. Wolke, Jennifer M. Phillips, Allan L. Reiss, Antonio Y. Hardan

**Affiliations:** 1 Department of Psychiatry and Behavioral Sciences, Stanford University, 401 Quarry Road, Stanford, CA 94305 USA; 2 Department of Psychiatry, University of Campinas, Cidade Universitária Zeferino Vaz, Campinas, SP 13083-970 Brazil; 3 Department of Biomedical Data Science, Stanford University, 1265 Welch Road, Stanford, CA 94305 USA; 4 Department of Anesthesiology, Stanford University, 300 Pasteur Drive, Stanford, CA 94305 USA

**Keywords:** Autism spectrum disorders, Neuroscience

## Abstract

Atypical growth patterns of the brain have been previously reported in autism spectrum disorder (ASD) but these alterations are heterogeneous across individuals, which may be associated with the variable effects of genetic and environmental influences on brain development. Monozygotic (MZ) and dizygotic (DZ) twin pairs with and without ASD (aged 6–15 years) were recruited to participate in this study. T1-weighted MRIs (*n* = 164) were processed with FreeSurfer to evaluate structural brain measures. Intra-class correlations were examined within twin pairs and compared across diagnostic groups. ACE modeling was also completed. Structural brain measures, including cerebral and cerebellar gray matter (GM) and white matter (WM) volume, surface area, and cortical thickness, were primarily influenced by genetic factors in TD twins; however, mean curvature appeared to be primarily influenced by environmental factors. Similarly, genetic factors accounted for the majority of variation in brain size in twins with ASD, potentially to a larger extent regarding curvature and subcortical GM; however, there were also more environmental contributions in twins with ASD on some structural brain measures, such that cortical thickness and cerebellar WM volume were primarily influenced by environmental factors. These findings indicate potential neurobiological outcomes of the genetic and environmental risk factors that have been previously associated with ASD and, although preliminary, may help account for some of the previously outlined neurobiological heterogeneity across affected individuals. This is especially relevant regarding the role of genetic and environmental factors in the development of ASD, in which certain brain structures may be more sensitive to specific influences.

## Introduction

Autism spectrum disorder (ASD) is a neurodevelopmental disorder that affects approximately 1 in 59 children in the United States [1]. ASD is characterized by cognitive/behavioral impairments in social communication (SCI) and restricted, repetitive patterns of behavior and interests (RRB) [2], with variable presentation and severity across individuals. The etiology of ASD is also variable with up to 20–25% of cases [3] arising from rare genetic abnormalities (e.g., single-gene disorders, chromosomal abnormalities, or copy number variation) but the vast majority likely stem from multifactorial genetic influences. In these cases, genetic vulnerability may interact with environmental influences [4] to alter the development of neuronal circuits [5]. As such, there have been reports of widespread neurobiological abnormalities in the brain in ASD [6]. However, these alterations are also variable. Thus, ASD is an inordinately heterogeneous disorder in terms of etiology, neurobiology, and symptomatology, which is likely associated with the effects of genetic and environmental interactions on different neurobiological pathways. The application of novel approaches, such as twin studies, may help to clarify the contribution of these components on brain development in ASD.

Twin studies provide an approach for estimating the influence of genetic versus environmental factors that contribute to a disorder. For instance, studies of monozygotic (MZ) and dizygotic (DZ) twin pairs suggest a high rate of genetic influence in ASD [7–10], with more recent studies indicating a potentially greater environmental contribution than previously reported [10]. The twin study design has also been applied in neurobiological investigations to assess the effects of genetic versus environmental factors that modulate neurodevelopment. Investigations of typically-developing (TD) twins suggest that brain volume (up to ~90%) [11, 12], surface area (71–89%), and cortical thickness (69–81%) [13, 14] are primarily influenced by genetic factors. This is relevant for the study of ASD because there are numerous reports of abnormal growth patterns of the brain, which indicate early overgrowth followed by a possible normalization later in life [15]. To date, the application of the twin design into neurobiological studies of ASD has been limited with most focusing on MZ twin pairs [16–18]. These studies largely corroborate reports of volumetric alterations in ASD, particularly regarding white matter (WM), and further suggest that cerebellar volume may be more influenced by environmental factors in ASD [16], see review [19]. These findings are informative regarding the neurobiological abnormalities that are associated with ASD, especially regarding the control for potential confounding sources of variability. However, these studies included relatively small sample sizes and did not compare MZ and DZ twins, which would help identify whether genetic or environmental factors are associated with the development of specific neurobiological differences.

In this investigation, we examine structural measures of the brain in MZ and DZ twin pairs with and without ASD to assess the influence of genetic and environmental factors on brain size. Based on previous investigations [11, 12, 16, 17], we hypothesized that cerebral tissue volume would be primarily influenced by genetic factors (i.e., MZ correlations would be significantly higher than DZ correlations) in both ASD and TD twins but that cerebellar volume, especially WM, would be influenced by environmental factors to a greater extent in ASD [16]. Surface area and cortical thickness are highly related to brain volume, but these components are genetically and phenotypically independent [13, 14] and exhibit different developmental trajectories [20, 21], suggesting that there may be differential contributions from genetic/environmental influences in ASD. Based on the relatively high estimates of environmental influences on gyrification in TD twins [22] and the lack of concordance in MZ ASD twin pairs [18], we also predicted that curvature of the brain would be largely influenced by environmental factors in both ASD and TD twin pairs. Examining the relationship between genetic and environmental interactions on the previously reported brain size differences in ASD will help elucidate the possible etiological pathways from which these differences arise. This could help account for more of the neurobiological heterogeneity across individuals. Furthermore, identifying the neurobiological pathways that are affected by environmental factors in ASD will improve neurodevelopmental models, which will be an important step towards increasing the potential for neurobiological stratification in the future.

## Methods and materials

### Participants

Ninety same-sex twin pairs (male/female) aged 6–15 years, in which at least one twin was diagnosed with ASD or both were TD, were recruited to participate in this study. Participants with ASD were initially identified from the California Autism Twin Study [10] (27%) and Interactive Autism Network Research Database (33%). Additional twin pairs with ASD and TD control twin pairs were recruited from local/online advertisements. Exclusionary criteria included any evidence of genetic/metabolic disorders, history of traumatic head injury or asphyxia at birth, unstable medical conditions, or magnetic resonance imaging (MRI) contraindication. Control participants were also excluded if there was history of learning disabilities or severe affective/psychiatric disorders (e.g., ASD, ADHD, schizophrenia, or major depression), as assessed with parent-report questionnaires, or a full-scale IQ (FSIQ) < 70, as assessed with the Stanford-Binet Intelligence Scales, Fifth Edition [23]. The Child Behavioral Checklist (CBCL) [24] was also collected and all TD twins exhibited *T*-scores ≤ 70 for Depressive, Anxiety, Somatic, Attention Deficit/Hyperactivity, Oppositional Defiant, Sluggish Cognitive Tempo, Obsessive Compulsive, and Stress Problems subscales, suggesting there were no clinically-relevant behavioral symptoms in our control group. For ASD twin pairs, clinical diagnosis was confirmed with the Autism Diagnostic Interview-Revised (ADI-R) [25] and Autism Diagnostic Observation Schedule, 2nd Edition (ADOS-2) [26], and participants were evaluated on an individual basis to determine his/her ability to complete study procedures. An initial power analysis that was based on 80 probands and 40 unrelated controls, allowing for 20% missing data, was completed with the STATA [27] program “powerlog” and indicated sufficient power to detect group-related differences within the expected effect size range.

Autism-related symptoms were compared between groups with the Social Responsiveness Scale (SRS) [28] and Short Sensory Profile (SSP) [29]. Handedness and socioeconomic status (SES) were assessed using the Edinburgh Handedness Inventory [30] and the Hollingshead method [31], respectively. Zygosity was confirmed from saliva samples based on nine short tandem repeat loci and the X/Y amelogenin. Concordance on all markers was considered MZ whereas discordance for at least one marker was considered DZ [10]. The methodology of the study was approved by the Institutional Review Board, and written informed consent was obtained from parents and assent from participants. Additional information on the participants and general study design was reported previously [32, 33].

### MRI acquisition and processing

MRI was conducted at two sites within the same institution, Lucile Packard Children’s Hospital and the Richard M. Lucas Center for Imaging, on identical GE 3T MR750 scanners (Waukesha, Wisconsin, USA) using a standard 8-channel head coil. For individuals with ASD that were unable to remain motionless, sedation with propofol was administered under the supervision of an anesthesiologist at a rate of 200–300 mcg/kg/min to induce light procedural sedation. Two T1-weighted IR SPGR echo pulse sequence images were acquired from each participant (188 coronal slices, TR = 8.15 ms, TE = 3.24 ms, inversion time = 600 ms, flip angle = 12 degrees, slice thickness = 1.2 mm, FOV = 22 × 22 cm, in-plane resolution = 0.86 × 0.86, and acquisition matrix size = 256 × 192 mm, NEX = 1), and the highest quality anatomical image was selected for further analysis.

Cortical reconstruction and volumetric segmentation was performed using FreeSurfer [34] (http://surfer.nmr.mgh.harvard.edu) and the Desikan-Killiany atlas [35]. Trained raters visually inspected all automated procedures and manually edited segmentations when errors were present. To evaluate potential site effects, two sets of twins (i.e., 4 participants) who did not require sedation were scanned at both locations. Repeated measures comparisons indicated an ~6% difference in total brain volume between sites. Affected scans (ASD and TD) were transformed, prior to segmentation, using the FSL linear transformation package FLIRT [36] with standard sinc interpolation to FSL standard orientation images. The transformation matrix (Supplemental Table S1) was designed to minimize our site-specific differences in brain volume, surface area, and thickness across all parameters. Global brain measures included volume estimates of cortical and cerebellar GM and WM, subcortical GM, the brainstem and ventricular volume (lateral + inferior lateral + 3rd + 4th ventricles). Total surface area and mean cortical thickness and curvature were also evaluated.

### Statistical analyses

Intra-class correlations (ICC), controlling for variation associated with gender and diagnosis, were first generated in all MZ and DZ twin pairs to examine general twin pair differences in structural brain measures and ensure that our data met the basic assumptions for twin modeling. Analyses were performed with STATA [27] under the DeFries-Fulker model [37] framework, which is not constrained by the assumptions of the ACE model [38]. ICCs within ASD and TD twin pairs, excluding those discordant for ASD, were then examined separately with the same approach and were compared between zygosity groups with Fisher’s z transformation to provide quantitative comparisons of twin pair differences between groups. Due to the sample size and basic analysis approach, discordant twin pairs were excluded from the diagnostic group specific analyses to remove the additional variability between twins with and without autism-related symptoms and increase generalizability to the greater ASD population. The ACE model for broad sense heritability was then calculated based on Falconer’s formula [39] to estimate the contribution of genetic and environmental factors on variation of structural brain measures. The ACE model estimates the proportions of variation in a trait of interest (e.g., structural brain measures) that are related to additive genetic factors (*a*^2^) and common/shared (*c*^2^) or unique (*e*^2^) environmental influences. Model components are generated by comparing trait variability in MZ versus DZ twin pairs, who share a common environment but differ in genetic influences by a known quantity (i.e., ~50%). ACE modeling was completed utilizing a bootstrapping method across 1000 repetitions. When A or C was non-significant, a simpler AE or CE model was utilized. Although diagnostic group comparisons were not the primary focus of the current investigation, comparisons between ASD and TD twins (Supplementary Table S2) and within twin pairs discordant for ASD (Supplementary Table S3) were included in the supplementary materials. In general, there were no major group differences in structural brain measures, especially after correction for multiple comparisons. These findings are not surprising because a relative “normalization” of global structural brain measures has been previously reported in individuals with ASD at similar developmental periods [15].

## Results

### Participants

T1-weighted images were acquired from 180 participants that comprised 90 twin pairs (55 ASD; 35 TD) (Table 1). Good quality scans were available from 30 twin pairs (15 MZ; 15 DZ) in which both twins had ASD, 18 twin pairs (4 MZ; 14 DZ) that were discordant for ASD, and 34 TD twin pairs (20 MZ; 14 DZ). Discordance for ASD was defined by one twin meeting diagnostic criteria on the ADI-R and ADOS whereas the other twin did not meet criteria for either ASD or the broader autism phenotype, in which sub-threshold ASD-related impairments are indicated. There were no group differences in age, sex, ethnicity, or handedness, *p* < 0.05 in all instances. Although SES was slightly lower in the ASD group, *p* = 0.047, primarily based on DZ twins, *p* = 0.016, there were no other zygosity by diagnostic group differences, *p* > 0.05. Additionally, adjusting for SES did not significantly alter the reported ICCs or subsequent modeling; thus, unadjusted estimates are discussed in more detail.

**Table 1:** Demographics and clinical characteristics of concordant twin pairs and twins with ASD and TD controls

Demographics	ASD			TD			ASD vs. TD		
Concordant twin pairs	MZ (*n* = 15)	DZ (*n* = 15)	All ASD (*n* = 30)	MZ (*n* = 20)	DZ (*n* = 14)	All TD (*n* = 34)	*t* or *χ*²	*p*	
Age (years)	11.07 (2.79)	10.73 (2.89)	10.90 (2.80)	10.05 (2.52)	8.93 (2.87)	9.59 (2.69)	1.91	0.06	
Sex (male/female)	11/4	13/2	24/6	15/5	9/5	24/10	0.75	0.39	
Ethnicity (A/B/H/W/MO)	1/0/1/10/3	1/1/2/9/2	2/1/3/19/5	1/0/1/15/3	1/0/0/12/1	2/0/1/27/4	3.27	0.51	
SES (Hollingshead)	53.25 (11.89)	47.40 (11.51)	50.22 (11.86)	54.25 (8.47)	57.46 (8.76)	55.52 (8.60)	−2.03	0.047*	d

Clinical characteristics									
Individuals	MZ (*n* = 34)	DZ (*n* = 44)	All ASD (*n* = 78)	MZ (*n* = 40)	DZ (*n* = 28)	All TD (*n* = 68)	*t* or *χ*²	*p*	
Handedness (right/left)	28/6	40/4	68/10	37/3	25/3	62/6	0.60	0.44	
Full scale IQ	83.97 (25.04)	79.55 (26.86)	81.53 (25.99)	110.98 (10.83)	115.86 (12.08)	112.99 (11.53)	−9.20	<0.001✝	c,d
Short sensory profile	143.21 (22.31)	142.51 (25.20)	142.79 (23.94)	174.29 (11.31)	176.24 (18.93)	175.10 (14.85)	−9.08	<0.001✝	c,d
Social responsiveness scale	72.97 (13.32)	69.45 (18.12)	71.03 (16.15)	45.35 (5.46)	41.07 (4.52)	43.63 (5.49)	13.23	<0.001✝	b,c,d
ADI-R diagnostic total	42.06 (12.89)	38.98 (15.64)	40.36 (14.46)	–	–	–	–	–	
ADOS-2 comparison score	7.44 (1.85)	7.23 (1.93)	7.32 (1.88)	–	–	–	–	–	

The current samples are segregated by zygosity, monozygotic (MZ) and dizygotic (DZ), and comprised of twin pairs in which both twins had autism spectrum disorder (ASD) or were typically-developing (TD) control twin pairs or all individuals with ASD and TD controls (ASD co-twins that were unaffected were not included). ADI-R diagnostic total is the sum of the social interaction, communication, and restricted/repetitive behavior. Group comparisons (ASD versus TD) were conducted with independent samples *t*-tests (*t*) or chi-squared (*χ*²). A/B/H/W/MO = Asian/Black/Hispanic/White/multiple or other. Significant group comparison at *p < 0.05 or ✝False Discovery Rate [73] corrected across the tests within each column.

a = MZ ASD versus DZ ASD *p* ≤ 0.05

b = MZ TD versus DZ TD *p* ≤ 0.05

c = MZ ASD versus MZ TD *p* ≤ 0.05

d = DZ ASD versus DZ TD *p* ≤ 0.05

As expected, twins with ASD exhibited more social deficits (based on the SRS), *p* < 0.001, sensory-processing abnormalities, *p* < 0.001, and lower FSIQ, *p* < 0.001, compared to TD twins, with generally no zygosity group differences within the ASD and TD samples, *p* > 0.05. The TD zygosity subgroups did exhibit a difference in total SRS scores, *p* < 0.001; however, both subgroups (SRS_MZ_ = 45.35, SD = 5.46; SRS_DZ_ = 41.07, SD = 4.52) were well below the threshold for clinically-relevant autism-related symptoms, which is between 65 and 70 depending on gender [40].

### Intra-class correlations (ICC) in MZ and DZ twin pairs

**Fig. 1:**
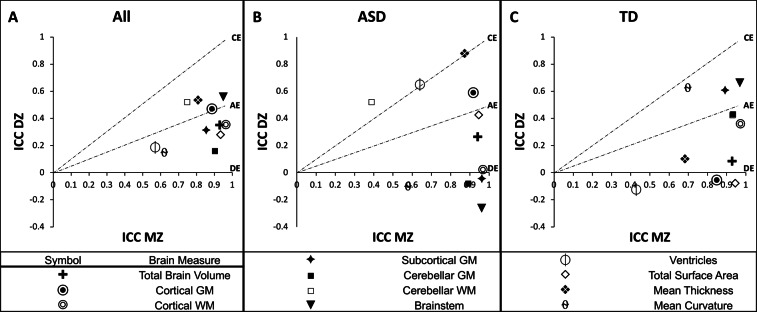
ICCs in ACE model space. Intra-class correlation coefficients (ICC) in **a** all participants, adjusted for diagnosis and gender, and separately within twin pairs in which both twins were diagnosed with **b** autism spectrum disorder (ASD) or were **c** typically-developing (TD) controls, adjusted for gender, were generated within monozygotic (MZ) and dizygotic (DZ) twin pairs and are displayed in relation to ACE model space [a = additive genetics; c = shared family environment; e = unique environment; d = genetic dominance]. Brain structures above or near the CE line are primarily environmentally-mediated whereas brain structures below or near the DE line are primarily genetically-mediated

Examining all twin pairs regardless of diagnosis, we found the anticipated pattern of ICC measures within and between zygosity subgroups, with ICCs generally positive, significant, and higher in MZ compared to DZ twins (Fig. 1a; Supplementary Table S4). The only exceptions were found in DZ twins in which cerebellar GM, *p* = 0.23, ventricular volume, *p* = 0.27, and mean curvature, *p* = 0.42, correlations were not statistically different than zero. However, previous studies have also reported relatively low magnitude correlations within DZ twin pairs for these brain measures [41] and the MZ twin pair ICCs were all still significantly higher, even after controlling for multiple comparisons. Thus, our data generally met the basic assumptions of twin modeling and all of aforementioned global brain measures were further examined in the ASD and TD subgroups.

Examining the ASD and TD twin pairs separately (Table 2; Fig. 1b, c) revealed salient ICC differences between diagnostic groups. All ICC estimates for MZ TD twins were statistically significant, *p* < 0.05 in all instances, and large magnitude (~ 0.70 to 0.95), with the exception of ventricular volume (*r*_MZ_ = 0.43, *p* = 0.024). All ICC estimates for MZ ASD twin pairs were also statistically significant, *p* < 0.05, with large magnitude correlations (~ 0.60 to 0.95) except for cerebellar WM (*r*_MZ_ = 0.39, *p* = 0.022), which was lower in MZ ASD compared to MZ TD twins (*r*_MZ_ = 0.93, *p* < 0.001), *p* < 0.001. In contrast, ICCs for subcortical GM (*r*_MZ_ = 0.96; *r*_MZ_ = 0.87; *p* = 0.029) and mean thickness (*r*_MZ_ = 0.89; *r*_MZ_ = 0.68; *p* = 0.043) were higher in MZ ASD compared to MZ TD twins, respectively. However, these differences did not survive correction for multiple comparisons.

**Table 2:** ICC comparisons between concordant ASD and TD twin pairs

	ASD				TD				ASD vs. TD			
Structure	MZ (*n* = 30)	*p*	DZ (*n* = 30)	*p*	MZ (*n* = 40)	*p*	DZ (*n* = 28)	*p*	*z* (MZ)	*p*	*z* (DZ)	*p*
Total brain volume	0.94 [0.77, 1.11]	<0.001✝	0.27 [−0.23, 0.77]	0.29	0.93 [0.83, 1.03]	<0.001✝	0.09 [−0.46, 0.64]	0.74	0.38	0.70	0.67	0.50
Cortical GM	0.92 [0.75, 1.09]	<0.001✝	0.60 [0.30, 0.90]	<0.001✝	0.84 [0.61, 1.06]	<0.001✝	−0.05 [−0.56, 0.46]	0.86	1.44	0.15	2.65	0.008✝
Cortical WM	0.97 [0.93, 1.01]	<0.001✝	0.03 [−0.63, 0.69]	0.93	0.97 [0.93, 1.02]	<0.001✝	0.37 [−0.15, 0.75]	0.06	−0.28	0.78	−1.28	0.20
Subcortical GM	0.96 [0.92, 1.01]	<0.001✝	−0.04 [−0.49, 0.42]	0.87	0.89 [0.74, 1.05]	<0.001✝	0.61 [0.28, 0.95]	<0.001✝	2.19	0.029*	−2.71	0.007✝
Cerebellum GM	0.89 [0.69, 1.10]	<0.001✝	−0.07 [−0.51, 0.37]	0.76	0.93 [0.87, 1.00]	<0.001✝	0.44 [0.02, 0.86]	0.039*	−0.99	0.32	−1.96	0.05
Cerebellum WM	0.39 [−0.23, 1.01]	0.22	0.53 [0.06, 1.01]	0.029*	0.93 [0.82, 1.05]	<0.001✝	0.44 [−0.11, 0.89]	0.06	−4.96	<0.001✝	0.45	0.65
Brainstem	0.96 [0.93, 0.99]	<0.001✝	−0.26 [−0.66, 0.14]	0.20	0.97 [0.95, 0.99]	<0.001✝	0.67 [0.28, 1.06]	0.001✝	−0.41	0.68	−3.85	<0.001✝
Ventricles	0.64 [0.45, 0.83]	<0.001✝	0.66 [0.26, 1.06]	0.001✝	0.43 [0.06, 0.80]	0.024✝	−0.12 [−0.50, 0.27]	0.55	1.19	0.23	3.26	0.001✝
Total surface area	0.95 [0.85, 1.04]	<0.001✝	0.43 [−0.04, 0.90]	0.07	0.92 [0.84, 0.99]	<0.001✝	−0.07 [−0.54, 0.40]	0.77	0.97	0.33	1.92	0.06
Mean thickness	0.87 [0.72, 1.02]	<0.001✝	0.89 [0.77, 1.00]	<0.001✝	0.68 [0.53, 0.84]	<0.001✝	0.11 [−0.24, 0.45]	0.54	2.02	0.043*	4.66	<0.001✝
Mean curvature	0.58 [0.34, 0.82]	<0.001✝	−0.10 [−0.57, 0.37]	0.69	0.70 [0.45, 0.94]	<0.001✝	0.63 [−0.10, 1.36]	0.09	−0.81	0.42	−3.02	0.003✝

Intra-class correlation coefficients (ICC), adjusted for gender, are compared with Fisher's *z* transformation between monozygotic (MZ) and dizygotic (DZ) twin pairs in which both twins were diagnosed with autism spectrum disorder (ASD) or were typically-developing (TD) controls. Twin pairs discordant for ASD were excluded. Total Brain Volume = all cortex (cortical gray matter (GM) and white matter (WM) + cerebellar GM/WM + brainstem + ventricles) and Ventricles = lateral + inferior lateral + 3rd + 4th. Significant correlation or group comparison at *p < 0.05 or ✝False Discovery Rate [73] corrected across the tests within each column are indicated

As expected, there was a much wider range of ICC estimates for both DZ TD (~ −0.10 to 0.70) and DZ ASD (~ −0.30 to 0.90) twins. Cortical GM (*r*_DZ_ = 0.60; *r*_DZ_ = −0.05; *p* = 0.008), ventricular volume (*r*_DZ_ = 0.66; *r*_DZ_ = −0.12; *p* = 0.001), and mean thickness (*r*_DZ_ = 0.89; *r*_DZ_ = 0.11; *p* < 0.001) showed higher ICCs in DZ ASD compared to DZ TD twin pairs, respectively. Additionally, ICCs for subcortical GM (*r*_DZ_ = −0.04; *r*_DZ_ = 0.61; *p* = 0.007), the brainstem (*r*_DZ_ = −0.26; *r*_DZ_ = 0.67; *p* < 0.001), and mean curvature (*r*_DZ_ = −0.10; *r*_DZ_ = 0.63; *p* = 0.003) were lower in DZ ASD compared to DZ TD twins.

### ACE modeling

The main comparisons of interest were between MZ and DZ twin pairs within diagnostic groups (Table 3), which provides group-specific estimates for the influence of genetic versus environmental factors. Within TD twins, all global brain measures, except mean curvature, were best fit with the AE model. *a*^2^ (additive genetic) estimates ranged from ~0.6 to 1, suggesting that genetic influences primarily contributed to global brain measures in TD twins. Conversely, mean curvature was best fit with the CE model. The *c*^2^ (common environment) estimate was 0.67, suggesting that curvature was the only global structural measure that was primarily influenced by environmental factors in TD twins.

**Table 3:** ACE models in concordant ASD and TD twin pairs

	A (additive genetics)						C (shared environment)						E (unique environment)			
Structure	ASD	CI	*p*	TD	CI	*p*	ASD	CI	*p*	TD	CI	*p*	ASD	CI	TD	CI
Total brain volume	0.84	[0.45, 1.14]	<0.001✝	0.81	[0.54, 1.07]	<0.001✝	–	–	–	–	–	–	0.16	[−0.14, 0.55]	0.19	[−0.07, 0.46]
Cortical GM	1.00	[0.77, 1.23]	<0.001✝	0.74	[0.46, 1.03]	<0.001✝	–	–	–	–	–	–	0.26	[−0.23, 0.23]	0.001	[−0.03, 0.54]
Cortical WM	0.79	[0.37, 1.21]	<0.001✝	0.93	[0.75, 1.11]	<0.001✝	–	–	–	–	–	–	0.21	[−0.21, 0.64]	0.07	[−0.11, 0.25]
Subcortical GM	×	×	×	0.95	[0.77, 1.13]	<0.001✝	–	–	–	–	–	–	×	×	0.05	[−0.13, 0.23]
Cerebellar GM	×	×	×	0.92	[0.73, 1.12]	<0.001✝	–	–	–	–	–	–	×	×	0.08	[−0.12, 0.27]
Cerebellar WM	–	–	–	0.92	[0.72, 1.12]	<0.001✝	0.48	[0.11, 0.85]	0.011✝	–	–	–	0.52	[0.15, 0.89]	0.08	[−0.12, 0.28]
Brainstem	×	×	×	1.04	[0.87, 1.21]	<0.001✝	–	–	–	–	–	–	×	×	−0.04	[−0.21, 0.13]
Ventricles	–	–	–	×	×	×	0.65	[0.46, 0.85]	<0.001✝	–	–	–	0.35	[0.15, 0.54]	×	×
Total surface area	0.93	[0.70, 1.16]	<0.001✝	×	×	×	–	–	–	–	–	–	0.07	[−0.16, 0.30]	×	×
Mean thickness	–	–	–	0.60	[0.40, 0.80]	<0.001✝	0.88	[0.80, 0.96]	<0.001✝	–	–	–	0.12	[0.04, 0.20]	0.40	[0.20, 0.60]
Mean curvature	×	×	×	–	–	–	–	–	–	0.67	[0.41, 0.92]	<0.001✝	×	×	0.33	[0.08, 0.59]

The ACE model for broad sense heritability was calculated separately for twin pairs in which both twins were diagnosed with autism spectrum disorder (ASD) or were typically-developing (TD) controls. Twin pairs discordant for ASD were excluded. When A or C was non-significant within the model (−), a constrained AE or CE model was utilized. When model assumptions were violated, model estimates were not generated, but the parameters that would have been most appropriate are indicated (×). Total brain volume = all cortex (cortical gray matter (GM) and white matter (WM) + cerebellar GM/WM + brainstem + ventricles) and Ventricles = lateral + inferior lateral + 3rd + 4th. E estimates were based on the residuals and were not tested for significance. Significant model parameter at *p < 0.05 and ✝False Discovery Rate [73] correction across the estimates within each column are indicated

Within ASD twin pairs, there were significant deviations from the ACE models that were found in TD twin pairs. Similar to TD twins, the majority of global brain measures in ASD twins were best fit with the AE model. *a*^2^ estimates ranged from ~0.8 to 1. However, cerebellar WM, ventricular volume and mean thickness were best fit with the CE model. *c*^2^ estimates ranged from ~0.5 to 0.9. Thus, there appeared to be a greater influence of shared environmental factors on structural brain measures in twins with ASD compared to TD control twins.

There were a few global brain measures that could not be fit with the ACE models, such as ventricular volume and total surface area in TD twins and subcortical GM and mean curvature in ASD twins. The most probable reason for these fitting errors was the presence of negative correlations in the DZ subgroups, which caused violations of modeling assumptions. Based on the comparatively larger ICCs in the MZ twins, it appeared that these brain measures were also primarily genetically-mediated, including mean curvature in ASD twin pairs.

## Discussion

In this investigation, brain size and other related global structural brain measures appeared to be primarily influenced by genetic factors in TD twins. The only exception was mean curvature, which was primarily associated with environmental factors. Similarly, genetic factors accounted for the majority of variation in brain size in twins with ASD, potentially to a larger extent for curvature and subcortical GM. However, there were also more environmental contributions to some brain structures in ASD. Cortical thickness and cerebellar WM volume were primarily influenced by environmental factors in ASD but not TD twin pairs. Cumulatively, these observations point to a possible increase in the vulnerability of certain brain structures to either genetic [3] or environmental [4] influences in individuals with ASD.

Findings from the present study are consistent with what has been previously reported in TD twin pairs [42], which indicated that brain size is primarily associated with genetic factors (66–97%) [11, 12, 22, 43–48], including cortical GM (65–82%) and WM (73–87%) [11, 12, 45–48], surface area (71–89%) [13, 14] and cortical thickness (69–81%) [13, 14, 45]. These estimates are similar to the current TD sample in which all brain measures that were assessed, excluding mean curvature, were also primarily influenced by genetic factors. Although some structural brain measures were not able to be evaluated with the ACE models, ICC comparisons between MZ and DZ twin pairs were in accordance with previous reports. Overall, these observations support the validity of our approaches and choice of control group.

Similar to the TD group, twins with ASD exhibited considerable genetic influences on global brain measures. Based on ICC comparisons between MZ and DZ twin pairs, total brain volume, cortical GM/WM, subcortical/cerebellar GM, the brainstem, and surface area, appeared to be primarily genetically-mediated in ASD, which was supported by ACE modeling for total brain volume, cortical GM/WM and surface area. These findings suggest that the aforementioned reports of macrocephaly/early brain size differences in individuals with ASD [15] may have been associated with genetic factors, which is consistent with reports of an association between brain size and altered gene expression profiles in ASD [49]. Some brain measures may have also been more genetically-mediated in twins with ASD. We observed a higher additive genetic estimate for cortical GM in ASD compared to TD twin pairs, but this could have been associated with the non-significant DZ correlations. Similarly, there were several brain measures that exhibited extremely high MZ correlations and extremely low DZ correlations in ASD twins, suggesting a potentially larger impact of genetic influences. However, statistically significant ICC differences across diagnostic groups, which would support clinically-relevant alterations, were mostly absent and the assumptions of ACE modeling were not met. Importantly, the potential genetic dominance effect that was observed for subcortical GM in twins with ASD was supported by diagnostic group comparisons of ICC estimates, which indicated significantly higher ICCs in MZ ASD compared to MZ TD twins and significantly lower ICCs in DZ ASD compared to DZ TD twins. Although previous investigations of TD twin pairs have reported considerable genetic influences on subcortical GM [43, 45, 46], those estimates were much lower than what was observed in the current ASD sample. Subcortical GM structures have also been previously implicated in ASD, with reports of larger volumes of the caudate [50, 51] and globus pallidus/putamen [52, 53] but smaller volume of the thalamus [54, 55]. Thus, genetic influences on brain size appear to be similar in children and adolescents with and without ASD, at least in terms of magnitude, but there are some structures, such as subcortical GM, that may be more heavily genetically-influenced in individuals with ASD.

In contrast to the other structural brain measures that were primarily genetically-mediated, mean curvature, which is associated with gyrification of the brain [56], appears to be primarily environmentally-mediated in TD twins. This finding was supported by both ICC comparisons and ACE modeling and is consistent with previous investigations that indicated gyral patterns are primarily influenced by non-genetic factors during typical development [22]. Conversely, curvature of the brain appeared to be more genetically-mediated in twins with ASD, based on ICC comparisons, albeit the DZ ICC did not reach significance and could not be modeled with the ACE approach. The development of gyrification patterns is partially driven by WM connections within neuronal circuits [57]. These “connectivity” patterns are primarily established during the third trimester of fetal development but continue to mature during postnatal development via synaptic pruning and dendritic arborization [58]. Environmentally-mediated changes in gyrification could reflect adaptive experience-based changes in neuronal connections. The finding of increased genetic influences on curvature in ASD may suggest that there is less adaptive experience-based alterations, which could be related to the reports of abnormal connectivity in ASD [59]. The only previous investigation of gyrification in twins with ASD reported ICCs that were lower (*~ *−0.58 to 0.15) [18] than the current investigation (−0.10 to 0.58). However, that sample was primarily comprised of discordant twin pairs, which likely reduced the magnitude. These observations highlight the complexity of ASD in which some brain structures may be more influenced by genetic factors, while others may be more impacted by environmental factors.

Twins with ASD also exhibited increased environmental influences on some structural brain measures, such as cortical thickness, which was supported by both ICC comparisons and ACE modeling. Cortical thickness is primarily genetically-mediated during typical development [13, 14, 45] but appeared to be primarily environmentally-mediated in the current ASD sample. Cortical thickness has been previously implicated in ASD [60, 61] with evidence suggesting a relationship with the aforementioned early overgrowth and later normalization. Existing evidence also indicates that surface area and cortical thickness are largely genetically-independent [13, 14]. Based on ICC comparisons, the current findings expand on this line of research and suggest that whereas surface area is primarily genetically-mediated in both TD and ASD twin pairs, cortical thickness may be influenced by environmental factors to a larger extent in ASD. The effects of environmental factors on altered developmental trajectories of cerebral size in children with ASD may exert their influence via pathways that modulate cortical thickness.

Additional findings from the current investigation also indicate the possible existence of increased environmental influences on cerebellar WM and ventricular volume in ASD. Previous estimates of genetic influences on cerebellar (49–88%) [12, 44, 46–48, 62] and ventricular (0–92%) [11, 12, 44, 46, 47, 63–65] volume in TD twins have been variable across investigations. The extreme variability regarding ventricular volume makes interpretation of potential ASD-related differences difficult, but estimates of genetic influences on cerebellar WM are still relatively high across studies. The magnitude of environmental influences on cerebellar WM in the current ASD sample are strikingly different than the current and previous estimates for TD twins for both ICC comparisons and ACE modeling estimates. This finding is also supported by the only previous investigation of cerebellar WM in twins with ASD [16], in which MZ twins concordant for ASD exhibited significantly higher ICCs compared to MZ discordant twin pairs. Cerebellar WM has also been previously implicated in ASD [66, 67]. Although these alterations are somewhat variable across investigations, prenatal loss of Purkinje cells in the cerebellum, which contribute axons to cerebellum WM, is consistently reported [68]. The cerebellum may also be particularly sensitive to the effects of environmental stressors during prenatal development [11, 69, 70], suggesting that environmental factors affecting the in utero environment may be associated with the development of volumetric abnormalities in this structure.

There are several limitations of the current investigation that should be considered. Our sample is rather large for an MRI study but was not large enough at times to apply more advanced twin modeling techniques, such as Defries-Fulker regression [37, 38]. This was especially evident in the DZ twin pairs in which non-significant and/or negative ICCs were found for several measures. The examination of twin pairs, which include related individuals, may have also affected the variability in our general diagnostic group comparisons. However, comparisons of only one twin from each pair were generally in accordance with the reported findings. The basic ACE model that was utilized assumes that shared environmental effects are the same for MZ and DZ twin pairs, which may allocate some environmental influences to the genetic factor, and also assumes there are no gene by environment interactions, which cumulatively may cause an overestimation of genetic influences. The concordance rates for ASD in the current sample were also somewhat higher compared to earlier estimates [7], especially for DZ twins, but were similar to recent findings from a more diverse population sample that was assessed with updated diagnostic criteria [10]. The two separate scanners that were utilized could have also introduced additional variability, so transformation of approximately one-third of the neuroimaging data was applied. Analyses excluding these data did not significantly differ from those reported for the full dataset. Finally, our findings indicate the magnitude with which genetic and environmental factors may influence brain size, not the specific factors that are driving these effects, and the global level measures provide information on the overall components (or compartments) that may play a role in the brain pathology of ASD but not the local contributing factors [46, 48, 71, 72].

In summary, brain size is primarily genetically-mediated during typical development, and our preliminary findings indicate a similar observation for individuals with ASD. However, genetic factors may influence subcortical GM to a larger extent in ASD, and environmental factors may exert a greater impact on the development of some brain structures, such as cortical thickness and cerebellar WM. Additional observations also indicated that individuals with ASD may undergo less adaptive environmentally-mediated changes in curvature/gyrification. Future investigations should assess larger twin samples to replicate these findings and additional efforts should be made to include more DZ twin pairs. Younger twin samples should also be evaluated to identify the genetic and environmental factors that influence brain structure during early development in ASD.

## Supplementary information

Supplementary Materials
