# Supplementary material for: Genetic and environmental influences on structural brain measures in twins with autism spectrum disorder
Source: Mol Psychiatry. 2019 Jan 18;25(10):2556–66. doi: 10.1038/s41380-018-0330-z (PMC6639158; doi:10.1038/s41380-018-0330-z)
Supplement: Supplementary file 1 — Supplementary Materials [file 41380_2018_330_MOESM1_ESM.docx]

**Supplementary Materials**

This supplement includes more detailed information and additional analyses for the investigation outlined in *Genetic and Environmental Influences on Structural Brain Measures in Twins Autism Spectrum Disorder,* including comparisons between twins with autism spectrum disorder (ASD) and typically-developing controls and within twin pairs discordant for ASD.

**Table S1. Transformation matrix.** To evaluate potential site effects, two sets of twins who were not sedated were scanned at both imaging locations, which indicated an ~6% difference in total brain volume across sites. Affected scans were transformed, prior to segmentation, using the FSL linear transformation package FLIRT with standard sinc interpolation to FSL standard orientation images.

| 0.97 | 0 | 0 | 0 |
| --- | --- | --- | --- |
| 0 | 0.975 | 0 | 0 |
| 0 | 0 | 0.98 | 0 |
| 0 | 0 | 0 | 1 |

| **Table S2. Group comparisons between twins with ASD and TD control twins.** | | | | | | | | | | |
| --- | --- | --- | --- | --- | --- | --- | --- | --- | --- | --- |
|  | ***ASD*** | | ***TD*** | | ***ASD vs TD*** | | ***ASD vs TD (adjust TBV)*** | | | ***Add. Comps*** |
| ***Region*** | M (n=78) | SD | M (n=68) | SD | t | p | F | | p |  |
| Total Brain Volume | 1251070.77 | 13388.69 | 1246437.50 | 14339.40 | 0.24 | 0.81 | - | | - | b,d |
| Cortical GM | 577772.78 | 6036.26 | 579646.52 | 6464.88 | -0.21 | 0.83 | 0.74 | | 0.39 | c |
| Cortical WM | 436855.63 | 6551.39 | 436970.87 | 7016.59 | -0.01 | 0.99 | 0.33 | | 0.56 |  |
| Subcortical GM | 63871.12 | 602.96 | 63254.19 | 645.78 | 0.70 | 0.49 | 0.69 | | 0.41 | b,d |
| Cerebellum GM | 124986.61 | 1350.52 | 122425.25 | 1446.41 | 1.29 | 0.20 | 2.46 | | 0.12 | b,d |
| Cerebellum WM | 27192.15 | 497.48 | 25882.09 | 532.80 | 1.80 | 0.07 | 4.18 | | 0.043* | d |
| Brainstem | 19838.70 | 260.37 | 19327.04 | 278.85 | 1.34 | 0.18 | 2.25 | | 0.14 | b,d |
| Ventricles | 15557.61 | 1000.71 | 13684.22 | 1071.77 | 1.28 | 0.20 | 1.60 | | 0.21 |  |
| Total Surface Area | 177237.67 | 1988.72 | 178574.41 | 2129.93 | -0.46 | 0.65 | 3.00 | | 0.09 |  |
| Mean Thickness | 2.83 | 0.01 | 2.82 | 0.01 | 0.52 | 0.61 | 0.28 | | 0.60 |  |
| Mean Curvature | 0.15 | 0.001 | 0.14 | 0.001 | 0.97 | 0.34 | 1.05 | | 0.31 |  |
| Group comparisons were completed with independent samples t tests and ANCOVA, adjusted for total brain volume (TBV), between twins with autism spectrum disorder (ASD) and typically-developing (TD) control twins (ASD co-twins that were unaffected were not included). Global brain measures were generated with FreeSurfer^1^ based on the Desikan-Killiany atlas.^2^ Total Brain Volume = all cortex (cortical gray matter (GM) and white matter (WM) + cerebellar GM/WM + brainstem + ventricles) and Ventricles = lateral + inferior lateral + 3rd + 4th. Significant comparison at *p < 0.05 or✝False Discovery Rate^3^ corrected across the tests within each column. | | | | | | | | | | |
| * = ASD vs TD p ≤ 0.05 |  |  | |  |  |  |  |  |  |  |
| a = MZ ASD vs. DZ ASD p ≤ 0.05 | |  | |  |  |  |  |  |  |  |
| b = MZ TD vs. DZ TD p ≤ 0.05 | |  | |  |  |  |  |  |  |  |
| c = MZ ASD vs. MZ TD p ≤ 0.05 | | | |  |  |  |  |  |  |  |
| d = DZ ASD vs. DZ TD p ≤ 0.05 | | | |  |  |  |  |  |  |  |

| **Table S3. Group comparisons within twin pairs discordant for ASD.** | | | | | | | | | |
| --- | --- | --- | --- | --- | --- | --- | --- | --- | --- |
|  | ***ASD*** | | ***Unaffected Co-twin*** | | ***ASD vs Co-twin*** | | ***ASD vs Co-twin (adjust TBV)*** | | ***Add. Comps*** |
| ***Region*** | M (n=18) | SD | M (n=18) | SD | t | p | F | p |  |
| Total Brain Volume | 1242999.11 | 138291.25 | 1277884.44 | 123619.04 | -1.37 | 0.19 | - | - |  |
| Cortical GM | 582047.11 | 50381.19 | 586106.00 | 50990.94 | -0.36 | 0.72 | 3.28 | 0.10 |  |
| Cortical WM | 430804.33 | 71096.71 | 452963.28 | 62933.17 | -1.83 | 0.09 | 2.33 | 0.15 |  |
| Subcortical GM | 63171.33 | 7102.95 | 65985.50 | 5373.14 | -1.89 | 0.08 | 0.30 | 0.59 |  |
| Cerebellum GM | 119851.34 | 14750.58 | 127778.89 | 11871.22 | -2.23 | 0.040* | 2.66 | 0.13 | d |
| Cerebellum WM | 25986.57 | 4743.91 | 26805.09 | 3435.09 | -1.05 | 0.31 | 0.01 | 0.92 |  |
| Brainstem | 19612.82 | 3086.91 | 20659.36 | 2245.87 | -2.73 | 0.014* | 5.69 | 0.034* | d |
| Ventricles | 16413.99 | 10567.74 | 13485.78 | 5626.00 | 1.63 | 0.12 | 3.04 | 0.11 |  |
| Total Surface Area | 176360.33 | 16553.11 | 181768.00 | 17648.33 | -1.38 | 0.18 | 0.40 | 0.54 |  |
| Mean Thickness | 2.86 | 0.11 | 2.80 | 0.11 | 2.81 | 0.012* | 9.84 | 0.009* | b,d |
| Mean Curvature | 0.14 | 0.01 | 0.15 | 0.003 | -0.88 | 0.39 | 6.36 | 0.027* | a |
| Group comparisons were completed with paired samples t tests and repeated measures ANCOVA, adjusted for total brain volume (TBV), within twin pairs in which the twins were discordant for autism spectrum disorder (ASD). Global brain measures were generated with FreeSurfer^1^ based on the Desikan-Killiany atlas.^2^ Total Brain Volume = all cortex (cortical gray matter (GM) and white matter (WM) + cerebellar GM/WM + brainstem + ventricles) and Ventricles = lateral + inferior lateral + 3rd + 4th. Significant comparison at *p < 0.05 or✝False Discovery Rate^3^ corrected across the tests within each column. | | | | | | | | | |
| * = ASD vs TD p ≤ 0.05 |  |  |  |  |  |  |  |  |  |
| a = MZ ASD vs. DZ ASD p ≤ 0.05 | |  |  |  |  |  |  |  |  |
| b = MZ TD vs. DZ TD p ≤ 0.05 | |  |  |  |  |  |  |  |  |
| c = MZ ASD vs. MZ TD p ≤ 0.05 | | |  |  |  |  |  |  |  |
| d = DZ ASD vs. DZ TD p ≤ 0.05 | | |  |  |  |  |  |  |  |

| **Table S4. ICC comparisons between all MZ and DZ twin pairs.** | | | | | | | |
| --- | --- | --- | --- | --- | --- | --- | --- |
|  | ***All Participants*** | | | | ***MZ vs DZ*** | | |
| ***Region*** | MZ (n=78) | p | DZ (n=86) | p | | z | p |
| Total Brain Volume | 0.94 [0.88,1.00] | <0.001✝ | 0.36 [0.10,0.61] | 0.006✝ | | 8.36 | <0.001✝ |
| Cortical GM | 0.88 [0.77,0.99] | <0.001✝ | 0.48 [0.28,0.67] | <0.001✝ | | 5.34 | <0.001✝ |
| Cortical WM | 0.97 [0.94,0.99] | <0.001✝ | 0.36 [0.03,0.70] | 0.035* | | 10.37 | <0.001✝ |
| Ventricles | 0.56 [0.36,0.77] | <0.001✝ | 0.19 [-0.15,0.54] | 0.27 | | 2.76 | 0.006✝ |
| Subcortical GM | 0.86 [0.72,1.01] | <0.001✝ | 0.32 [0.05,0.60] | 0.022✝ | | 6.13 | <0.001✝ |
| Cerebellum GM | 0.91 [0.85,0.97] | <0.001✝ | 0.17 [-0.11,0.45] | 0.23 | | 8.40 | <0.001✝ |
| Cerebellum WM | 0.75 [0.48,1.03] | <0.001✝ | 0.53 [0.30,0.76] | <0.001✝ | | 2.41 | 0.016✝ |
| Brainstem | 0.97 [0.95,0.98] | <0.001✝ | 0.56 [0.29,0.83] | <0.001✝ | | 8.66 | <0.001✝ |
| Ventricles | 0.56 [0.36,0.77] | <0.001✝ | 0.19 [-0.15,0.54] | 0.27 | | 2.76 | 0.006✝ |
| Total Surface Area | 0.93 [0.88,0.99] | <0.001✝ | 0.29 [0.04,0.53] | 0.022✝ | | 8.66 | <0.001✝ |
| Mean Thickness | 0.81 [0.70,0.91] | <0.001✝ | 0.54 [0.32,0.76] | <0.001✝ | | 3.19 | 0.001✝ |
| Mean Curvature | 0.62 [0.46,0.78] | <0.001✝ | 0.15 [-0.22,0.53] | 0.42 | | 3.54 | <0.001✝ |
| Intra-class correlation coefficients (ICC) are compared with Fisher’s z transformation between monozygotic (MZ) and dizygotic (DZ) twin pairs for all participants, adjusted for diagnosis and gender. Global brain measures were generated with FreeSurfer^1^ based on the Desikan-Killiany atlas.^2^ Total Brain Volume = all cortex (cortical gray matter (GM) and white matter (WM) + cerebellar GM/WM + brainstem + ventricles) and Ventricles = lateral + inferior lateral + 3rd + 4th. Significant correlation or group comparison at *p < 0.05 and✝ False Discovery Rate^3^ correction across the tests within each column. | | | | | | | |

**REFERENCES**

1. Fischl B. FreeSurfer. *Neuroimage* 2012; **62**(2)**:** 774-781.

2. Desikan RS, Ségonne F, Fischl B, Quinn BT, Dickerson BC, Blacker D *et al.* An automated labeling system for subdividing the human cerebral cortex on MRI scans into gyral based regions of interest. *NeuroImage* 2006; **31**(3)**:** 968-980.

3. Benjamini Y, Hochberg Y. Controlling the false discovery rate: a practical and powerful approach to multiple testing. *Journal of the Royal Statistical Society* 1995; **57**(1)**:** 289-300.
